# DisArticle: a web server for SVM-based discrimination of articles on traditional medicine

**DOI:** 10.1186/s12906-017-1596-4

**Published:** 2017-01-28

**Authors:** Sang-Kyun Kim, SeJin Nam, SangHyun Kim

**Affiliations:** 1 Mibyeong Research Center, Korea Institute of Oriental Medicine, 1672 Yuseong-daero, Yuseong-gu, Daejeon, 34054 Republic of Korea; 2 National Center of Excellence in Software, Chungnam National University, 99 Daehak-ro, Yuseong-gu, Daejeon, 34134 Republic of Korea

**Keywords:** Traditional medicine, Northeast Asia, MEDLINE, Support vector machine, Trend analysis

## Abstract

**Background:**

Much research has been done in Northeast Asia to show the efficacy of traditional medicine. While MEDLINE contains many biomedical articles including those on traditional medicine, it does not categorize those articles by specific research area. The aim of this study was to provide a method that searches for articles only on traditional medicine in Northeast Asia, including traditional Chinese medicine, from among the articles in MEDLINE.

**Results:**

This research established an SVM-based classifier model to identify articles on traditional medicine. The TAK + HM classifier, trained with the features of title, abstract, keywords, herbal data, and MeSH, has a precision of 0.954 and a recall of 0.902. In particular, the feature of herbal data significantly increased the performance of the classifier. By using the TAK + HM classifier, a total of about 108,000 articles were discriminated as articles on traditional medicine from among all articles in MEDLINE. We also built a web server called DisArticle (http://informatics.kiom.re.kr/disarticle), in which users can search for the articles and obtain statistical data.

**Conclusions:**

Because much evidence-based research on traditional medicine has been published in recent years, it has become necessary to search for articles on traditional medicine exclusively in literature databases. DisArticle can help users to search for and analyze the research trends in traditional medicine.

**Electronic supplementary material:**

The online version of this article (doi:10.1186/s12906-017-1596-4) contains supplementary material, which is available to authorized users.

## Background

MEDLINE is a bibliographic database that includes metadata and citations of biomedical literature. Although it covers articles in varying areas, including medicine, pharmacy, and biology from around the world, it does not categorize those articles by specific research area. The bibliographic content on MEDLINE is manually indexed through MeSH (Medical Subject Headings) [1], and the content can be searched for a specific topic using MeSH in Pubmed. However, because MeSH was originally designed to index, catalogue, and search articles with a controlled vocabulary thesaurus, it is difficult to apply it to the classification of academic disciplines.

Traditional medicine, particularly in Northeast Asia including traditional Chinese medicine (TCM), has developed from ancient times. A number of evidence-based articles have been published in this area in recent years. While MEDLINE also contains articles on traditional medicine, it does not offer a way to search for traditional medicine articles exclusively, making it difficult for researchers to analyze research trends in traditional medicine. Traditional medicine articles are often classified by MeSH headings such as “Medicine, Chinese Traditional”, but many articles remain without such a classification. Particularly in traditional medicine, a number of studies are being conducted in relation to herbal drugs, and these studies are generally classified by MeSH headings such as “Drugs, Chinese Herbal”. However, because studies on the effects of extracts or genomes of herbs are often identified by MeSH headings of “Plant Extracts” and “Genes, Plant”, respectively, it is not sufficient to use MeSH to determine whether the article is about traditional medicine. Therefore, in order to search for articles on traditional medicine, it is necessary to search for articles not only using MeSH, but also additional keywords. However, because different keywords will bring different search results, it is difficult to search exclusively for traditional medicine articles.

In academic disciplines, there generally exist journals that mainly publish articles for a specific discipline. However, all of the articles in the discipline are not always published in the given journal and literature databases such as MEDLINE include many journals covering various areas. Therefore, it is difficult to discriminate articles on traditional medicine from those of other disciplines by using only the journal information.

In order to overcome these difficulties, this research devised a classifier to identify articles on Northeast Asian traditional medicine by using the Support Vector Machine (SVM), which is widely used in text mining. We also constructed a web server called DisArticle, in which only articles on traditional medicine can be searched for from among all articles in MEDLINE. The major goal of DisArticle was to reduce the workload of researchers by reducing the number of articles they search and identify. This can help them to easily analyze research trends in traditional medicine.

Much research on machine learning techniques has been done, such as classification based on the MEDLINE database. The research on classification mainly has been done to discover new knowledge such as protein-protein interactions [2] or gene disease associations [3]. This research has been also used to extract gene terms [4] or chemical names [5] within the content of articles. Recently, an SVM-based classifier was constructed to determine whether a certain article describes a randomized clinical trial (RCT) [6]. MEDLINE not only includes the article publication type of the RCT, but also defines what work is about the RCT (http://www.ncbi.nlm.nih.gov/mesh/68016449). However, because the identification of RCTs is conducted in a simple way, this study proposes a classifier model to identify RCT articles using only the metadata and MeSH terms of each article.

## Implementation

### Data preparation

The corpus used to establish a classifier to identify traditional medicine articles was prepared directly from MEDLINE, with PubMed identifiers (PMID) ranging from 22,000,000 to 22,240,000. To increase the recall of the classifier, we only selected articles with both a title and an abstract, leading to a total of 189,674 articles. After the texts were extracted from the titles, abstracts, keywords, affiliation, journal name, and MeSH, they were indexed using Apache Lucene [7] to search for the features of the classifier. Two researchers in traditional medicine then manually reviewed the articles to determine whether they were about traditional medicine. The classification criteria were the biomedical research in relation to the terms within the WHO International Standard Terminologies (IST) [8], or medical herbs listed in the pharmacopoeia of China, Korea, and Japan. The WHO IST was constructed to provide a standardized nomenclature of traditional medicine in the Western Pacific region including China, Korean, and Japan. The nomenclature includes technical terms such as theories, diagnostics, therapeutics, acupuncture, and moxibustion. Because WHO IST excludes herbal drugs, we extracted the names of herbs from the Chinese, Korean, and Japanese government-published pharmacopeias. Cohen’s kappa for inter-rater agreement was 0.96, which is considered as substantial agreement. The two reviewers finally selected 1,537 traditional medicine articles after reaching a consensus. This paper used those articles as a gold standard for our classifier.

### Training and testing methods

This research used WEKA [9] to identify articles on traditional medicine in the MEDLINE database. A Java language-based software developed by the University of Waikato, WEKA provides not only a range of machine learning algorithms for data mining, but also a number of tools for data pre-processing, classification, and visualization. In particular, WEKA implements the Sequential Minimal Optimization (SMO) algorithm [10] to train the SVM classifier.

**Fig. 1:**
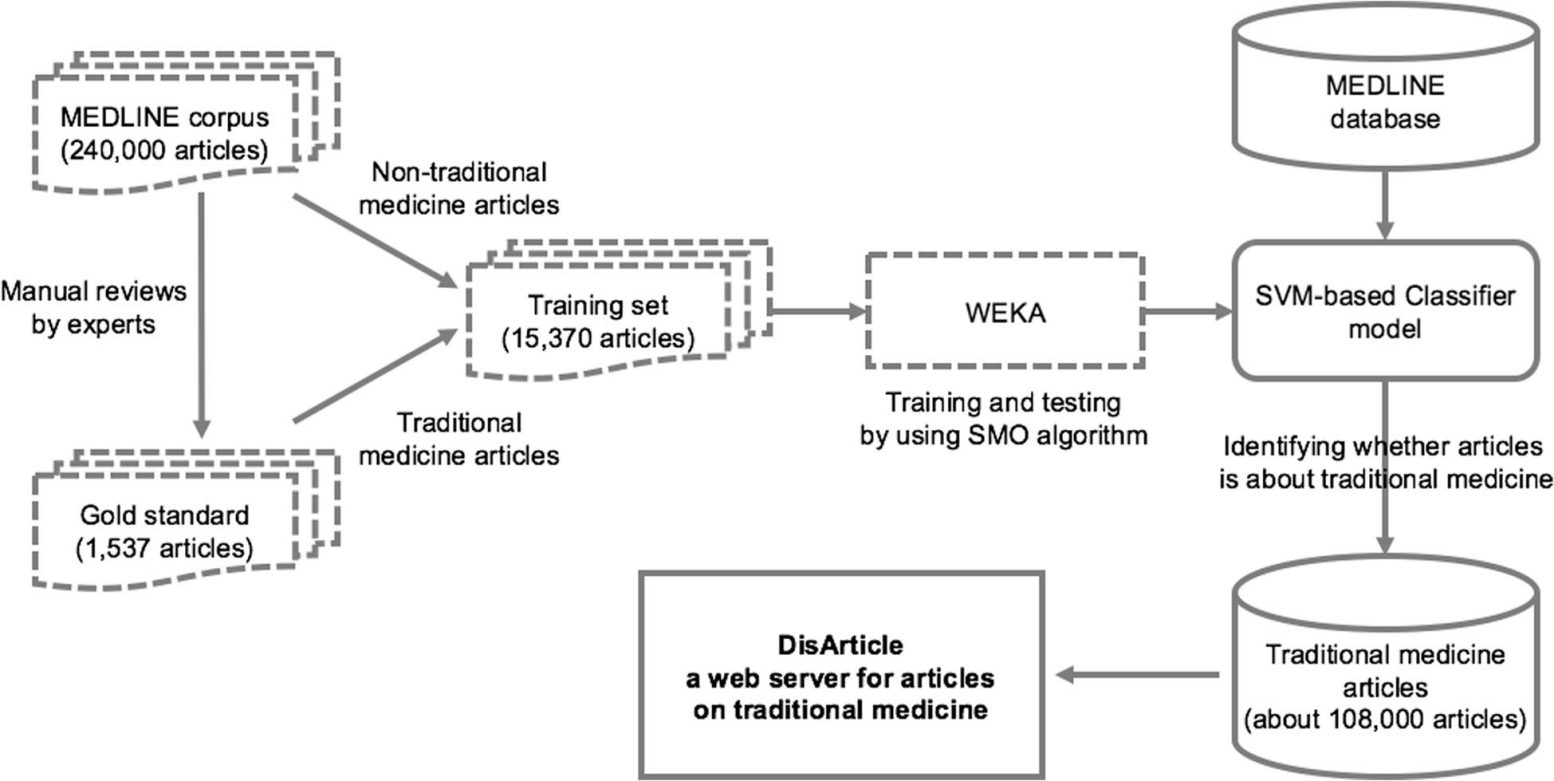
Process of implementation of our web server

The number of articles on traditional medicine is very small compared to the total number of articles in MEDLINE. Thus, for the training set used for the classifier model, gold standard articles and additional articles in quantity nine times the number of gold standard articles were randomly selected from the PMID range mentioned above, producing a total of 15,370 articles. The features for the training of the classifier included the title, abstract, keywords, affiliation, journal title, MeSH, and herbs. The herbal data consisted of the Latin, common, and scientific names of herbs in the Korean, Chinese, and Japanese pharmacopoeia as well as the medicinal parts of these herbs. The herbal feature is a binary value to determine whether such data exist in the title and abstract of each article. For the features other than herbs, the StringToWordVector filter provided by WEKA was used to create a vector for the words frequently found in each feature. The SMO algorithm used attributes, consisting of both the binary value of the herbs and the word vector as the kernel input, and finally classified all article instances as 1 or 0. The SVM classifier was trained with a polykernel with the default parameters provided in Weka and was tested with 10-fold cross validation. Figure 1 shows the overall process for the implementation of our web server, in which the stages of the solid line boxes are described in the results section.

### Performance measure

The objective of our classifier is to discriminate articles on traditional medicine from those of other disciplines. Using the articles on traditional medicine as the gold standard, the terminology used to evaluate of our classifier are follows:

- True positive (TP) – an article is identified as a traditional medicine article by both the classifier and the gold standard.
- True negative (TN) – an article is not identified as a traditional medicine article by either the classifier or the gold standard.
- False positive (FP) – an article is identified as a traditional medicine article by the classifier but not by the gold standard.
- False negative (FN) – an article is identified as a traditional medicine article by the gold standard but not by the classifier.

Precision, recall, and F-measure are calculated in the usual way:

- Precision = TP/(TP + FP)
- Recall = TP/(TP + FN)
- F-measure = (2 * Precision * Recall)/(Precision + Recall)
- Accuracy = (TP + TN)/(TP + TN + FP + FN)

## Results

### Classifier model

Because our classifier uses many attributes to identify traditional medicine articles, the number of attributes affects the classifier performance. Table 1 shows the performance of identifying traditional medicine articles with varying numbers of attributes created for all features. In the results, the ALL-140 classifier model with 140 attributes was observed to have the highest F-measure, and the level of performance dropped when the number of attributes was too big or small. The list of attributes and the detailed accuracy of the ALL-140 classifier model are given in the Additional file 1.

**Table 1:** Performance of classifiers by number of attributes (The value of the number in the name of the classifiers indicates the number of attributes used in that classifier)

	Precision	Recall	F-Measure	Accuracy
ALL-40	0.927	0.886	0.906	0.982
ALL-90	0.955	0.88	0.916	0.984
ALL-140	0.952	0.91	0.93	0.986
ALL-190	0.951	0.903	0.926	0.986
ALL-240	0.949	0.904	0.926	0.985
ALL-290	0.948	0.899	0.923	0.985
ALL-340	0.945	0.902	0.923	0.985

Table 2 shows the performance of the classifier models with different combinations of features. The number of attributes was maintained at 140, which value showed the highest performance, as can be seen in Table 1. The ALL-140 classifier had the best performance with an F-measure of 0.93. While the best precision obtained was 0.957 when using the TAK + H classifier excluding affiliation, journal title, and MeSH, the recall of the classifier was relatively small compared to ones that used herbal data (H). In general, a classifier with more features tended to show better performance. In particular, when the feature of herbal data was added, the performance increased significantly, which indicates that herbal data is an important feature in identifying articles on traditional medicine. While the feature of MeSH also increases the performance, herbal data is a more important feature than MeSH in the performance. It was shown that the performance of the TAK-AJM classifier excluding only herbal data is lower than that of TAK-H using only herbal data. For this reason, we do not include the performance of all combinations of features without herbal data in Table 2.

**Table 2:** Performance of the classifiers by feature (ALL: all attributes, TAK: title, abstract, keyword, H: herbs, A: affiliation, J: journal, M: MeSH)

	Precision	Recall	F-Measure	Accuracy
TAK	0.858	0.593	0.702	0.950
TAK + M	0.887	0.682	0.771	0.959
TAK + AJM	0.879	0.71	0.785	0.961
TAK + H	0.957	0.862	0.907	0.982
TAK + HA	0.953	0.889	0.92	0.985
TAK + HJ	0.954	0.893	0.923	0.985
TAK + HM	0.954	0.902	0.927	0.986
TAK + HAJ	0.95	0.897	0.923	0.985
TAK + HAM	0.95	0.908	0.928	0.986
TAK + HJM	0.952	0.903	0.927	0.986
ALL-140	0.952	0.91	0.93	0.986

### Web server

Articles on traditional medicine were discriminated from among those registered on MEDLINE with titles and abstracts up to January 8, 2016. Using the TAK + HM classifier model, a total of about 108,000 articles were identified as being about traditional medicine. Although the model test showed the best F-measure for the ALL-140, this result was not significantly better than that of the TAK + HM. Moreover, because precision is generally more important than recall in a web server used for searching [11], we chose the TAK + HM classifier model, which has higher precision, for our web server. The highest precision was found with the TAK + H; however, we did not choose this classifier because its recall is relatively small. The list of attributes and the detailed accuracy of the TAK + HM classifier model are provided in the Additional file 1.

With the article database established in this manner, this study built a web server called DisArticle in which users can search for articles on traditional medicine. DisArticle also enables users to identify other articles besides those in MEDLINE and to obtain statistical data such as the top ten researched herbs and the top ten journals publishing the most articles on traditional medicine. All these functions are provided at <http://informatics.kiom.re.kr/disarticle>. The main menu of this web server consists of Distinction, Search, and Statistics.

The Distinction menu enables users to identify whether an article is on traditional medicine. When a user submits the PMID of a certain article or its metadata, the distinction result is shown. Because distinction is also possible with the metadata of an article, any article that has not been identified by our web server can be identified in this menu. The web server also provides the identification API, so that a number of articles can be identified at the same time. In the Search menu, users can search for articles already identified as traditional medicine articles in our web server. When the keyword is inputted into the search field and the “Search” button is clicked, a list of articles matching the keyword is shown. In order to provide the annual distribution, the search results are also given as a bar chart, in which the x axis is the year and y axis the number of articles. The Statistics menu currently provides two sorts of statistical data about articles on traditional medicine. One is the annual distribution of articles on the top ten researched herbs among all traditional medicine articles, and the other is the annual distribution of the top ten journals that publish the most articles on traditional medicine. A detailed explanation of the use of our web server is given in the Help menu, and a full PMID list of the gold standard used in our classifier can be downloaded from the Introduction menu of our web server.

**Fig. 2:**
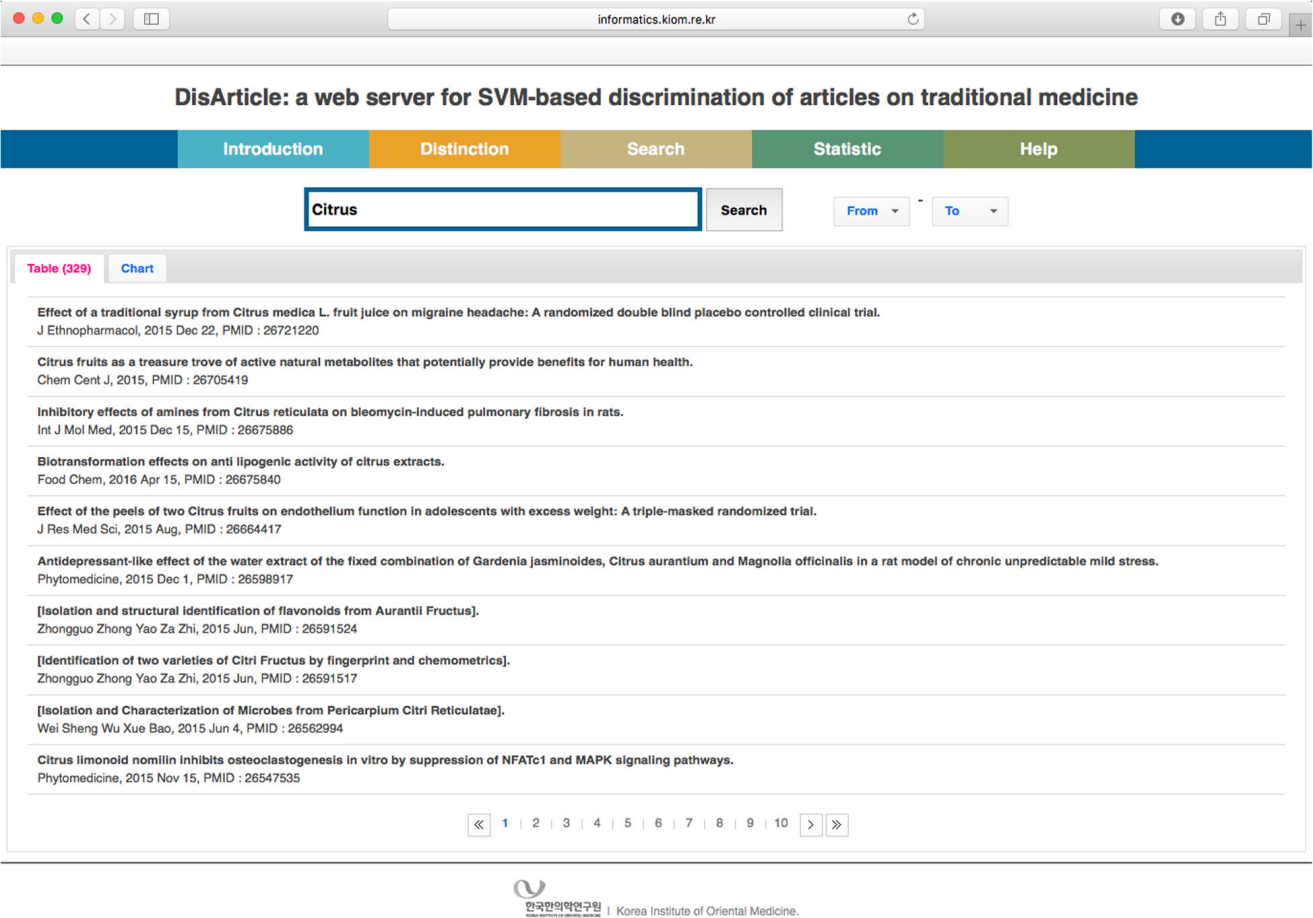
Result of search for the keyword “Citrus”

Figure 2 shows an example of a search for articles with respect to *Citrus* species in our web server. *Citrus unshiu* Markovich and *C. reticulata* Blanco are *Citrus* species known as “Jinpi” (Korean) or “Chenpi” (Chinese). In Northeast Asia including China, Korea, and Japan, the dried peels of these species have been used since ancient times as traditional herbal drugs for the treatment of gastrointestinal and inflammatory diseases [12–14]. Recently, as the peeled fruit is known as a rich source of flavonoids and carotenoids, several studies have been done with respect to the edible tissue of citrus fruits [15]. Therefore, if the keyword “Citrus” is searched in Pubmed, many articles with respect to the whole fruit including the peel and the pulp of *C. unshiu* Markovich and *C. reticulata* Blanco are listed. However, our web server can show only research on citrus fruits used in traditional herbal drugs.

## Discussion

The efficacy of traditional medicines from Northeast Asia, including TCM, has been proven clinically since ancient times. With the development of modern medicine, a number of published studies have shown scientific evidence on the efficacy of traditional medicine. What significantly distinguishes traditional medicine in Northeast Asia from other regional traditional medicines, such as traditional African medicine or Ayurveda medicine, is mainly the use of herbal drugs [16]. Certain medicinal herbs are only grown in Northeast Asia, and the same herbs may produce different medicinal effects depending on the region where they are grown. This research showed that herbal data are an important feature for identifying articles on traditional medicine. Therefore, our classifier should be trained with herbal data from other regions to provide broader coverage of other traditional medicines from around the world.

Recently, because of the increasing global interest in healthcare, a number of studies on complementary and alternative medicines (CAMs) are being done. CAMs are generally known as any medicinal practice that does not originate from scientific evidence. The CAM category includes TCM and other herbal medicines, as well as non-traditional medicines [17]. In order to build a web server to identify articles on CAMs, it is of foremost necessity to define criteria that can be used to show whether a certain article is about a CAM. This study used the WHO IST and the pharmacopoeia of Korea, China, and Japan as the criteria to identify articles on Northeast Asian traditional medicine. If the criteria for the identification of CAM articles is defined, it will be possible to establish an article identification system for CAMs similar to our web server.

As stated in the background, the work of Cohen et al. is similar to ours. We both constructed SVM-based binary classifiers for articles on MEDLINE and provided methods to determine whether an article is in a particular research area or of a particular type. Cohen et al. used only metadata and MeSH to identify articles. However, to achieve good performance, identifying articles in the traditional medicine field requires more features such as those that describe medicinal herbs.

In future work, we will do experiments with not only the SVM but also with a variety of machine learning algorithms. In addition, although the web server currently provides only basic statistical data on articles on traditional medicine, it will be updated to provide more professional trend analysis [18] or meta-analysis [19] data about traditional medicine articles.

## Conclusions

DisArticle provides a method to search for articles on Northeast Asian traditional medicine. In order to construct DisArticle, we first devised an SVM-based classifier model to identify articles on traditional medicine. The classifier model was made to discriminate between articles on traditional medicine and those of other disciplines for all articles that have both titles and abstracts in MEDLINE. DisArticle makes it possible for researchers to search for articles on traditional medicine, helping them to understand annual trends in traditional medicine research.

## Additional file

Additional file 1: Information on the ALL-140 and TAK-HM classifier model. (DOCX 97 kb)

## Abbreviations

CAM: Complementary and alternative medicine
DisArticle: Discrimination of articles
MeSH: Medical subject headings
PMID: PubMed identifier
RCT: Randomized clinical trial
SMO: Sequential minimal optimization
SVM: support vector machine
TCM: traditional Chinese medicine
WHO IST: World Health Organization International Standard Terminologies
