# Supplementary material for: DisArticle: a web server for SVM-based discrimination of articles on traditional medicine
Source: BMC Complement Altern Med. 2017 Jan 28;17:77. doi: 10.1186/s12906-017-1596-4 (PMC5273838; doi:10.1186/s12906-017-1596-4)
Supplement: Additional file 1: — Information on the ALL-140 and TAK-HM classifier model. (DOCX 97 kb) [file 12906_2017_1596_MOESM1_ESM.docx]

**Supplementary data**

**1. List of attributes used in ALL-140 and TAK-HM classifier model**

For the features other than the herbal data, the StringToWordVector filter provided by WEKA was used to create a vector for the words frequently found in each feature. The followings are attributes, consisting of the word vector in ALL-140 and TAK-HM classifier model.

**(1) ALL-140**

acid activity administration adult adverse age aged analysis animals based blood cancer care cell cells center chemistry clinical compared complications conclusions control data de department development diagnosis disease diseases dna drug effect effects epidemiology expression factors female found gene genetic genetics group groups growth health high higher hospital human humans immunology increased induced institute journal levels low male medical medicine metabolism method methods mice middle model models molecular pathology patient patients pharmacology physiology protein proteins rate related research response results risk role school showed significant significantly specific studies study surgery system therapeutic therapy time treatment tumor type university usa years activities acupuncture agents anti apoptosis china chinese chromatography compounds conclusion dose drugs extract extracts factor herbal inflammatory inhibitors isolation kg liquid liver medicinal mg ml pain plant plants potential proliferation purification rats sciences species total traditional treated vitro zhongguo

**(2) TAK+HM**

acid activity administration adult adverse age aged analysis animals based blood cancer care case cases cell cells chemistry clinical compared complications conclusion conclusions control data development diagnosis disease diseases dna drug effect effects epidemiology expression factor factors female found function gene genetic genetics group groups growth health high higher human humans immunology increased induced level levels low male metabolism method methods mice middle model models molecular observed outcome pathology patient patients pharmacology physiology potential present protein proteins rate rats related response results risk role showed significant significantly specific studies study surgery system therapeutic therapy time total treatment tumor type years activities acupuncture agents anti apoptosis chinese chromatography compounds decreased dose drugs extract extracts herbal inflammatory inhibited inhibitors isolation kg liquid liver medicinal medicine mg ml objective pain plant plants proliferation purification regulation serum species stress traditional treated vitro

**2. Results of training and testing for the ALL-140 and TAK-HM classifier model**

=== Run information ===

Filter : StringToWordVector- W100-prune-rate-1.0-N0-L-S-stemmerweka.core.stemmers.SnowballStemmer-M1-tokenizerweka.core.tokenizers.AlphabeticTokenizer

Classifier : SMO -C 1.0 -L 0.001 -P 1.0E-12 -N 0 -V -1 -W 1 -K "weka.classifiers.functions.supportVector.PolyKernel -C 250007 -E 1.0"

**(1) ALL-140**

Correctly Classified Instances 15160 98.6337 %

Incorrectly Classified Instances 210 1.3663 %

Kappa statistic 0.9226

Mean absolute error 0.0137

Root mean squared error 0.1169

Relative absolute error 7.5886 %

Root relative squared error 38.9629 %

Total Number of Instances 15370

=== Detailed Accuracy By Class ===

TP Rate FP Rate Precision Recall F-Measure ROC Area Class

0.995 0.09 0.99 0.995 0.992 0.952 0

0.91 0.005 0.952 0.91 0.93 0.952 1

Weighted Avg. 0.986 0.082 0.986 0.986 0.986 0.952

=== Confusion Matrix ===

a b <-- classified as

13762 71 | a = 0

139 1398 | b = 1

**(2) TAK+HM**

Correctly Classified Instances 15152 98.5817 %

Incorrectly Classified Instances 218 1.4183 %

Kappa statistic 0.9192

Mean absolute error 0.0142

Root mean squared error 0.1191

Relative absolute error 7.8777 %

Root relative squared error 39.6981 %

Total Number of Instances 15370

=== Detailed Accuracy By Class ===

TP Rate FP Rate Precision Recall F-Measure ROC Area Class

0.995 0.098 0.989 0.995 0.992 0.948 0

0.902 0.005 0.954 0.902 0.927 0.948 1

Weighted Avg. 0.986 0.089 0.986 0.986 0.986 0.948

=== Confusion Matrix ===

a b <-- classified as

13766 67 | a = 0

151 1386 | b = 1
